# A Pilot Study Using Next-Generation Sequencing in Advanced Cancers: Feasibility and Challenges

**DOI:** 10.1371/journal.pone.0076438

**Published:** 2013-10-30

**Authors:** Glen J. Weiss, Winnie S. Liang, Michael J. Demeure, Jeff A. Kiefer, Galen Hostetter, Tyler Izatt, Shripad Sinari, Alexis Christoforides, Jessica Aldrich, Ahmet Kurdoglu, Lori Phillips, Hollie Benson, Rebecca Reiman, Angela Baker, Vickie Marsh, Daniel D. Von Hoff, John D. Carpten, David W. Craig

**Affiliations:** 1 Virginia G. Piper Cancer Center Clinical Trials at Scottsdale Healthcare (VGPCC), Scottsdale, Arizona, United States of America; 2 The Translational Genomics Research Institute, Phoenix, Arizona, United States of America; 3 Van Andel Research Institute, Grand Rapids, Michigan, United States of America; Duke-National University of Singapore Graduate Medical School, Singapore

## Abstract

**Purpose:**

New anticancer agents that target a single cell surface receptor, up-regulated or amplified gene product, or mutated gene, have met with some success in treating advanced cancers. However, patients' tumors still eventually progress on these therapies. If it were possible to identify a larger number of targetable vulnerabilities in an individual's tumor, multiple targets could be exploited with the use of specific therapeutic agents, thus possibly giving the patient viable therapeutic alternatives.

**Experimental Design:**

In this exploratory study, we used next-generation sequencing technologies (NGS) including whole genome sequencing (WGS), and where feasible, whole transcriptome sequencing (WTS) to identify genomic events and associated expression changes in advanced cancer patients.

**Results:**

WGS on paired tumor and normal samples from nine advanced cancer patients and WTS on six of these patients' tumors was completed. One patient's treatment was based on targets and pathways identified by NGS and the patient had a short-lived PET/CT response with a significant reduction in his tumor-related pain. To design treatment plans based on information garnered from NGS, several challenges were encountered: NGS reporting delays, communication of results to out-of-state participants and their treating oncologists, and chain of custody handling for fresh biopsy samples for Clinical Laboratory Improvement Amendments (CLIA) target validation.

**Conclusion:**

While the initial effort was a slower process than anticipated due to a variety of issues, we demonstrate the feasibility of using NGS in advanced cancer patients so that treatments for patients with progressing tumors may be improved.

## Introduction

Patients with advanced cancer often exhaust treatment options. They may participate in Phase I or Phase II trials of new anticancer agents if they meet typically strict eligibility criteria and have access to centers that can administer investigational agents. When patients participate in these trials, new agents, on average, give response rates of between 5% and 10% in a Phase I setting and 12% in a Phase II setting [1]–[3]. Patients also have an option for best supportive care in an attempt to address their symptoms.

Recently, there has been an explosion of interest in developing new anticancer agents that are more targeted, usually against a cell surface receptor or an up-regulated or amplified gene product or mutated gene. This approach is meeting with some success (e.g. trastuzumab against HER2/*neu* in breast cancer cells, erlotinib against EGFR-mutant non-small cell lung cancer, etc.). However, patients' tumors still eventually progress on these therapies because they contain multiple genomic abnormalities, and targeting a single abnormality is not sufficient to prevent progression. If it were possible to identify a larger number of targets in an individual's tumor where there exist agents that could potentially target them, multiple targets could be addressed using specific therapeutic agents, and perhaps reduce the chance of progression. Ultimately, most investigators envision utilizing several agents to hit multiple targets present in a patient's tumor. However, identification and application of the appropriate therapeutics remains a challenge.

We previously conducted a prospective multicenter study utilizing molecular profiling of tumors by immunohistochemistry (IHC), fluorescent in situ hybridization (FISH), and DNA microarray to find potential drug targets and selected treatments accordingly [4]. Sixty-six of 84 patients were treated based on molecular profiling of their tumor. For 18 of these 66 patients, the treatment derived by molecular profiling, led to a progression-free survival ratio ≥1.3, thereby suggesting a treatment benefit. Molecular profiling supported the indication of a new treatment not contemplated initially by the investigator, in a patient population that was heavily pretreated and refractory to previous treatments.

To build upon this initial step towards personalized therapy, we used next-generation sequencing technologies (NGS) including whole genome sequencing (WGS), and where feasible, whole transcriptome sequencing (WTS) to identify genomic events and associated expression changes in advanced cancer patients. We used WGS to sequence tumor biopsy DNA and matched germline DNA from nine advanced cancer patients to identify key somatic changes. The germline DNA was sampled from white blood cells and the tumor DNA was sampled from tumor cells. For six of these patients, we also used WTS to sequence total RNA isolated from the tumor, along with non-patient total RNA controls. Because gene expression profiles differ between tissue types, and healthy tissue could not be biopsied from the patient for comparison, commercially purchased normal RNA for the corresponding tissue was compared to RNA isolated from the tumor. We then evaluated transcriptomic changes and performed integrated genomics analyses [5] with WGS data to identify potential druggable targets. Here, we demonstrate the feasibility and highlight the challenges of using NGS technologies in a prospective manner in advanced cancer patients.

## Methods

### Ethics Statement

The study was conducted in accordance with the Declaration of Helsinki and was approved by the Western Institutional Review Board (WIRB® Protocol #20101288)(NCT01443390). Written informed consent was obtained from patients, including written consent for publication of the clinical details and images.

### Study Objectives

The primary objective of this study was to identify as many genomic changes as possible in advanced cancers, so as to expand the range of potential actionable targets with therapies that were commercially available or clinical trials. The secondary objective was to develop a workflow process from tumor biopsy to treatment; indeed, this process must occur in a short enough timeframe in order for patients to benefit from this additional information in developing a treatment plan. This included measuring the time from biopsy to completion and final analysis of NGS on patient tumor and non-tumor samples, examining the frequency with which useable sequence data is obtained as a function of percent tumor involvement in the biopsy, and assessing the correlation between anti-tumor activity of treatments identified by NGS.

### Study Design

The rationale for this study is that NGS could be used to identify not just one but many genomic abnormalities that could be targeted using potentially available therapies in advanced cancer patients. This single center, prospective, single-arm pilot study was conducted in patients with advanced cancer that progressed on standard systemic therapy or if his or her tumor type did not have a standard systemic therapy. This study was exploratory in nature. To participate, patients must have been age ≥18 and willing to undergo a biopsy or surgical procedure to obtain tissue, unless a frozen tumor collected less than 8 weeks prior was available. Interested participants were made aware that obtaining a new biopsy may not be a part of the patient's routine care for their malignancy. Other eligibility criteria included: baseline laboratory data indicating acceptable bone marrow reserve, liver, and renal function, Karnofsky performance status ≥80%, and life expectancy >3 months. Participation on another clinical trial involving treatment prior to or during participation on this study was allowed. Main exclusion criteria were symptomatic or untreated central nervous system (CNS) metastases, known active infections requiring intravenous antimicrobial therapy, pregnant or breast-feeding women, or tumor that was inaccessible for a biopsy. Individuals with known HBV, HCV, or HCV infection(s) requiring antiviral therapy were also excluded as this population is often excluded from first-in-human oncology clinical trials; thus limiting the scope for potential treatment options for this group. All eligible patients had whole blood and a fresh frozen tumor sample collected and sent for analyses. These methods and information on raw sequencing data are described further in the Supplementary section. We initially anticipated approximately 100 days from the fresh tumor biopsy to completion of NGS. After NGS analysis was performed, the principal investigators reviewed the results, and when clinically appropriate, chose targets for validation by a CLIA-certified (Clinical Laboratory Improvement Amendments) laboratory. For five patient samples with low tumor percentage, exome sequencing was added to identify potential actionable targets. After target validation, a report was provided to the treating oncologist with potential treatment recommendations, which may be used at his/her discretion.

## Results

### Patient Characteristics

All patients were seen and evaluated for inclusion in this study between October 2010 and February 2012 at a single center. To avoid work flow delays on available NGS machines, screening for patients was spaced to allow no more than two potential candidates a month. As the center conducting the study receives referrals from throughout the country for phase 1 studies, some individuals came from out of state for a scheduled screening and consent evaluation, and after confirmation of eligibility, proceeded with a fresh tumor biopsy within 24 hours. This was done for patient convenience to allow for travel back home shortly after the initial assessment. It did create logistical challenges with follow-up and conveying results back to the treating physician. For patients living within driving distance, the enrollment process was similar, though the biopsy was not always scheduled for the next day.

Table 1 lists the characteristics of the 11 patients who consented for this study. The majority of patients were men, with a median age of 59 years (range, 20 to 69 years). Of note, at the time of consent, all but one patient (patient 9) had progressed on at least one prior systemic therapy for advanced disease (3, range 0–8). Patient 9, diagnosed with advanced adenosquamous carcinoma of the pancreas, underwent systemic therapy and subsequently progressed after NGS results became available.

**Table 1:** Sequencing outcomes.

Patient #	Tumor type	Gender	Age at Consent	# Systemic therapies prior to consent for NGS	% Tumor	Germline coverage	Tumor coverage	Whole transcriptome sequencing performed?	# Targets	# FDA-approved drugs (drug class)	# Investigational compounds (drug class)	Time for sequencing/analysis (days)	Challenges
Patient 1	Metastatic liposarcoma	M	59	2	0%	N/A	N/A	N	N/A	N/A	N/A	N/A	A, D
Patient 2* [9]	Metastatic neversmoker lung adenocarcinoma	F	61	3	60%	22	20	Y	0	0	0	124	C, D, E
Patient 3** [10]	Metastatic adenocarcinoma of pancreas	M	57	3	40%	43	54	N	1	0	1	91	C, D
Patient 4*** [11]	Metastatic olfactory neuroblastoma	M	29	1	50%	71	68	N	1	1	0	141	D
Patient 5	Metastatic thymic carcinoma	M	65	2	0%	N/A	N/A	N	N/A	N/A	N/A	N/A	A, D
Patient 6	Metastatic sertoli cell carcinoma	M	30	3	30%	47	55	N	1	0	1	243	B, D, F
Patient 7	Metastatic basal cell carcinoma of the skin	M	47	8	50%	22	18	Y	3	1	2	82	B, D
Patient 8**** [12]	Metastatic transitional cell urothelial cancer	F	20	2	30%	55	46	Y	1	1	0	152	B, D
Patient 9	Advanced adenosquamous pancreas cancer	M	69	0	Not available	29	20	Y	3	1	2	46	None
Patient 10	Metastatic papillary renal cell carcinoma	M	59	4	50%	24	26	Y	5	3	2	63	B
Patient 11	Metastatic bronchial neuroendocrine cancer	F	68	5	Not available	21	23	Y	3	3	0	67	B, G
		**Median:**	59	3	40%	29	26	-	1	1	1	91	
		**Range:**	(20, 69)	(0, 8)	(0%, 60%)	(21, 71)	(20, 68)	-	(0, 5)	(0, 4)	(0, 2)	(46, 243)	

M-male; F-female; N/A-not applicable; Y-yes; N-no; A-Insufficient DNA for sequencing; B-Low % tumor. Exome sequencing data were generated and used in identification of targets. For the protocol used, exome sequencing required more input DNA than WGS and provided information about exons and flanking untranslated regions of the genome; C-Patient expired or entered hospice care before sequencing data could be generated and analyzed; D-Unable to validate target(s) in a clinical laboratory setting because tissue was handled outside such a setting from start of study. Targets implicated by WGS data should be validated using alternative means, such as capillary sequencing. Validation is ideally performed in compliance with federal regulatory standards for clinical laboratory testing; E-No targets identified; F-Low % tumor. Targets initially identified based on WGS data could not be validated; G-Targets identified from RNA sequencing only;

* -Preliminary NGS results reported [12];

** -Full NGS results reported [13];

*** -Full NGS results reported [14];

**** -Preliminary NGS results reported [15].

**Figure 1:**
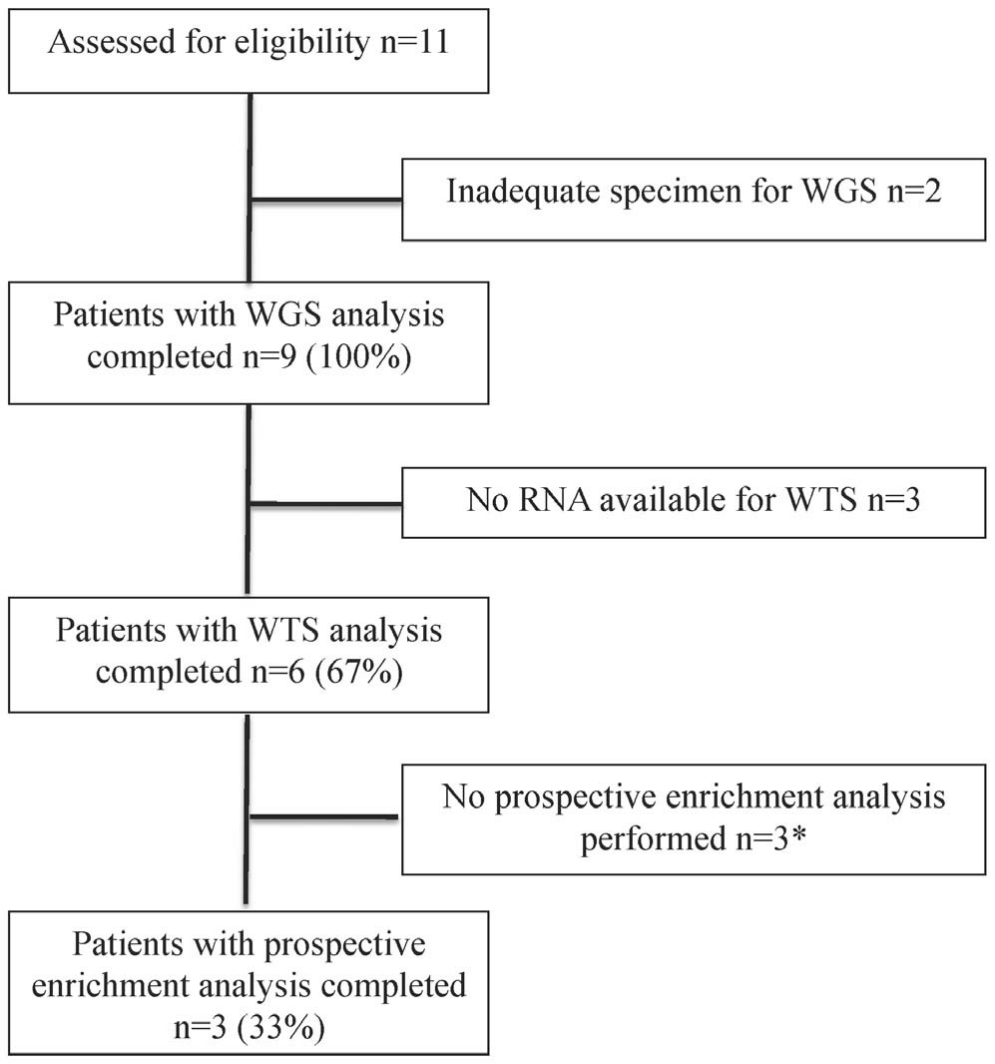
CONSORT schematic on patient enrollment. *-Prospective enrichment analysis was performed prior to discussing the results with the patient and treating oncologist for the last three patients enrolled.

Figure 1 details the Consolidated Standards of Reporting Trials (CONSORT) diagram demonstrating the flow of the 11 patients who consented and were evaluated for the study. Two patients (patients 1 and 5) underwent tumor biopsy and had insufficient DNA available for NGS analysis, because of lack of adequate tumor cellularity observed in the collected specimens (Table 1 and Table S1). For the remaining nine patients with advanced cancer, we performed WGS on both tumor DNA, as well as germline DNA isolated from whole blood in order to identify both somatic changes; respectively. WGS metrics and summary statistics for each of the nine patients are shown in Table S1. Six patients had available tumor RNA that were successfully analyzed by WTS. For the last three patients (patients 9, 10, and 11) enrolled on this study, we undertook a more involved biological analysis of the assembled genomic data to discern notable biological pathways that may be affected in the patient's cancer to identify possible therapeutic targets.

Of the 9 patients with WGS and/or exome sequencing and/or WTS analysis available, we identified potential targets and pathways for all but patient 2 (88.9%) (Table 1). For our secondary objectives, the median time from biopsy to completion and final analysis of NGS on patient tumor and non-tumor samples was 91 days (range 46–243 days). The main reasons for the outliers were either sequencing instrument problems or a result of low tumor percentage in the specimen requiring exome sequencing to identify potential actionable targets. For the protocol used, exome sequencing required more input DNA (3 ug) than WGS (1 ug) and provided information about coding regions and flanking untranslated regions of the genome. For seven of the cases, the initial tumor samples were submitted directly to our institution for pathology assessment so that tumor percentage could be ascertained. The minimum tumor cellularity content for successful NGS was 30% (Table 1).

### Whole genome sequencing

For the complete pilot study, sequencing was performed by synthesis technology and 100 bp paired-end chemistry. We generated over 19 billion total reads from WGS for average mapped coverages ranging from 17× to 71×. SNP (single nucleotide polymorphism) calling was performed using two separate callers to reduce the false negative rate. To evaluate the overall quality of variant data, germline SNPs were called and the transition to transversion and dbSNP (Single Nucleotide Polymorphism Database) [6] 129 concordance ratios were calculated. For all patients, the transition/transversion ratios were in the range of 2.01 to 2.24, and the dbSNP 129 concordance rate ranged from 86.0 to 89.4 (Table S1). From these analyses no biases were detected with respect to nucleotide substitutions, the SNPs identified strongly correlate with common genetic variations, and high quality variant calling was performed. For all patients there were a total of 5,778 coding genomic events identified (median = 15.5). Patient 2, a female never-smoker with metastatic lung adenocarcinoma, harbored the majority of these coding genomic events (n = 4,137) including a nonsynonymous TP53 mutation (G226V) with 100% mutant allele specific expression, and an interstitial 8q CNV (copy number variation) gain encompassing MYC. Patient 11, a female with metastatic bronchial carcinoid, had the fewest coding genomic events (n = 4). Exome sequencing was performed on tumor samples from patients 6, 7, 8, 10, and 11 with metrics provided in Table S1. CNV results are provided in Table S2.

### Whole transcriptome sequencing

WTS was performed in six patients (Table 1 and Table S1) and corresponding non-patient normal tissue RNA based on the primary tumor's origin. Because normal adjacent tissue was not collected from patients during biopsy, total RNA for the relevant tissue was commercially purchased. RNA libraries were prepared for these samples and sequenced with respective tumor RNA libraries. Tumor WTS data was compared to corresponding normal tissue WTS data to identify expression changes in the tumor biopsies. Each of the analyzed tumors had genes with significant expression changes with q<0.05, corrected for multiple testing (median number of genes differentially expressed 1,731, range 495–2,323)( Table S3).

### Copy Number Variation

Amplifications and deletions were detected by identifying regions where there was a delta (+/−) in log2FC (as described above) greater than 2 standard deviations the median log2FC across the arm of the chromosome being interrogated. Delta was calculated by subtracting the end-points of approximately 1 MB sliding window. In addition, the detected regions needed to be less then 14 MB in length to be marked as a focal event. Table S2 contains all focal events which contain COSMIC genes for Patients 2, 3, 4, 7, and 8. Patients 9 and 11 did not have any focal events that contained COSMIC genes. Patients 6 and 10 did not have any focal events (see Table S3 and Figure S7)

### Prospective Enrichment Analysis

For the last three patients enrolled, we performed enrichment analysis of WGS and/or exome sequencing and WTS. Details for each individual patient are as follows:

### Patient 9 adenosquamous carcinoma of the pancreas

Of the nine patients that had tumors analyzed by NGS, patient 9 (adenosquamous pancreas cancer) is the only individual whose treatment was based on targets and pathways identified by NGS. Only two types of data were available for analysis on this patient's tissues, single nucleotide variations (SNV) and WTS. No significant regions of alteration were observed in copy number data.

The top 20 canonical maps enriched in the WTS data are displayed in Figure S1. Common biological mechanisms associated with these top 20 enriched maps include adhesion, cytoskeletal remodeling and immune/chemotaxis processes. Such enrichments imply a generalized activation of extracellular matrix remodeling that may be related to phenotype for this rare tumor type. Two maps of note in the top twenty pathway map enrichments are specific pancreatic cancer maps built specifically to capture processes altered in pancreatic cancer. These maps titled, ‘Tumor-stroma interactions in pancreatic cancer’ and ‘Role of stellate cells in progression of pancreatic cancer’, further highlight possible stromal/ECM interplay within this tumor. The ‘Role of stellate cells in progression of pancreatic cancer’ map is presented in Figure S2.

An additional observation from inspection of overlaid WTS data onto signaling maps is the possible involvement of TGF-beta mediated epithelial to mesenchymal transition in this tumor. The ‘Development Regulation of epithelial to mesenchymal transition (EMT)’ map (Figure S3), highlights the WTS data overlaid as represented by the thermometers next to nodes in diagram. A red thermometer signifies that the gene is overexpressed and blue signifies that the gene is underexpressed.

Two ligands, TGF-beta 1 and TGF-beta 2 are both upregulated along with their cognate receptor, TGF-beta receptor type II. The combined upregulation of growth factors/receptor pairs points to potential importance of this signaling pair of being ‘active’ in this tumor. Additional evidence linking TGF beta signaling to EMT is highlighted by the upregulation of Lef-1, TCF8 and E2A transcription factors. These transcription factors function downstream of TGF beta to signal for EMT. Further evidence of their activation is the down-regulation of E-cadherin which is a hallmark of the EMT phenotype. The presence of the EMT phenotype would portend relative resistance to many therapeutic agents and signify a metastatic tumor.

Further inspection of the WTS data reveals the overexpression of possible targets for intervention. As mentioned above, ligand/receptor pair regulation suggests activation and upregulation of Neuropilin 1 and VEGF-A. This may represent vulnerabilities to anti-angiogenic therapies. Additional targets that were upregulated are ESR1, ABL1, GART, VDR and ERBB2.

Patient 9's tumor had notable mutations in KRAS (G12R) and PIK3CA (R93W). Both of these mutations activate signaling for growth and proliferation. These coexistent mutations suggest that combined targeting of MEK/ERK and PI3K/AKT for treatment of this tumor would be more effective than targeting just one of the two mutations. While the KRAS mutation was validated by CLIA testing, the region containing the PIK3CA mutation was not part of a commercial test and thus unconfirmed under CLIA conditions. Another prominent SNV is in the gene RAD50 (Q737R), which codes for a protein that is involved in DNA double-strand break repair. Defective function of RAD50 has been linked to sensitization to cisplatin and increased sensitivity to PARP inhibitors [7].

**Figure 2:**
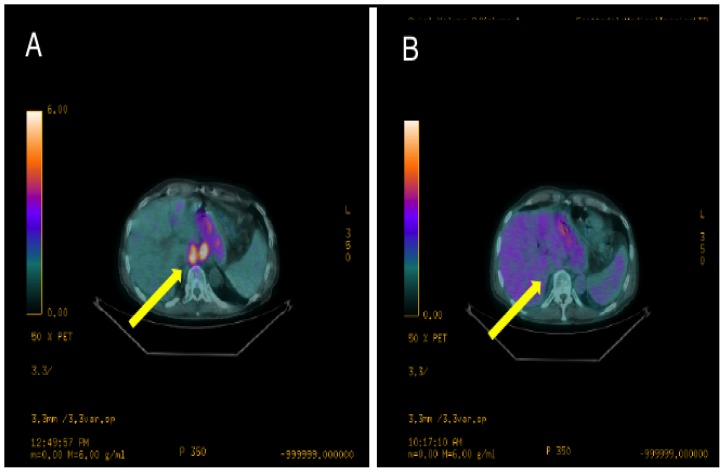
Patient 9 PET/CT images. 18-fluorodeoxyglucose positron emission tomography/computed tomography (PET/CT) images depicting (A) axial slice at baseline and (B) axial slice after 30 days on treatment. The yellow arrow is pointing to two hypermetabolic lymph nodes in B and C. In B, the standard uptake value (SUV) of the left lymph node is 9.8 and the lymph node on the right is 7.5, while the SUV of the left lymph node is 3.1 and the lymph node on the right is 3.5 in C.

The patient was initially treated with cisplatin and gemcitabine and then progressed. At that time the WGS/WTS results became available and the patient enrolled onto a phase I PI3K inhibitor and MEK inhibitor combination study. The patient had a short-lived PET/CT response on this study (Figure 2) and this was also accompanied by a dramatic decrease in his pain from moderate to none (4/10 to 0/10 on the visual pain scale).

### Patient 10 metastatic papillary renal cell carcinoma

Data for analysis for this patient consisted only of SNVs and WTS as no copy number aberrations were identified. The top 20 canonical maps reveal common themes around cell cycle, chromosome/spindle function, cell adhesion, and EMT transition. One pathway of note is the ‘Cell cycle spindle assembly and chromosome separation’ map (Figure S4). A number of genes involved with this map are upregulated in this tumor, including the druggable target AURKB (log2ratio = 6.3). Spindle assembly and chromosome separation are vital for cell division with a number of oncology drug targets associated with this process. Two SNVs are also present in genes (STAG1 (F802Y) and NUMA1 (L1400P)) in this pathway and suggest the importance of these alterations in this tumor.

Other pathways with statistical significance that contain important observations relating to the biology of this tumor and to possible therapeutic options are noted. The ‘EGF- and HGF-dependent stimulation of metastasis in gastric cancer’ map revealed upregulation of HGF and its receptor MET. This map was constructed from gastric cancer specific information, though many cancers share similar signaling pathways and molecules. Having both receptor and ligand upregulated suggests an autocrine signaling mechanism. As shown in Figure S5, alpha-6/beta-4 integrin components are upregulated and function in concert with MET to activate downstream signal transduction. The possible involvement of MET is an important observation as it is currently an attractive oncology drug target.

Additional inspection of data revealed the presence of alterations at the RNA level of potential drug targets. The angiogenic signaling ligand VEGFA, TOP2A, and ASNS mRNAs are upregulated. These three genes are targets of current oncology agents. Additionally, MGMT mRNA, which is a DNA repair gene that conveys resistance to temozolomide, is downregulated, suggesting temozolomide as a treatment option. Lastly, this tumor has a SNV in the gene APTX that encodes for a protein involved in DNA repair that could convey sensitivity to irinotecan [8].

### Patient 11 metastatic bronchial neuroendocrine cancer

Three types of genomic information available for analysis from this patient's tissues included WTS, CNV (Table S2), and SNV data. Only four SNVs in two genes, KRT4 and GOLGA6L10, were identified, resulting in the WTS and CNV data driving the biological narrative on this tumor. Figure S6 displays the top 20 canonical pathway maps from enrichment analysis of patient 11's data. The themes represented by the titles of these maps are not as concise as in patient 10, but again, we observe cytoskeleton and adhesive maps dominating. Of note, no cell cycle or chromosome centric maps are enriched possibly suggesting a minimally mitotic tumor. In terms of gene targets, there were several specific overexpressed genes including KIT, PDGFRB, EGFR, FGFR1 and SPARC. It should be noted that no copy number variations were seen in these genes.

## Discussion

In this exploratory pilot study, we built upon our prior experience with prospective molecular profiling. As this is an emerging technology, there were expected and unforeseen challenges encountered during the course of the study conduct. The population of patients evaluated in this pilot had advanced cancers with disease progression and unfortunately, for two individuals, the results became available after significant clinical deterioration or death (patients 2 and 3). While the study was ongoing, the cost of sequencing continued to decrease, and improved tools for WTS and integration of WGS with WTS emerged. Thus, WTS was added prospectively from patient 7 onward. For patient 2, WTS was feasible as there was available tumor for RNA extraction. The additional information from WTS for patient 2 was then relayed to the treating oncologist. Besides insufficient tumor DNA (patients 1 and 5), the technology and workflow of sample processing improved where we were able to incorporate exome sequencing (patients 6, 7, 8, 10, and 11) when tumor content percentage was inadequate from the sample biopsy. This added to the total sequencing and analysis time for these patients (Table 1) [9]–[12]. Note that WGS was the preferred modality during the conduct of this study. WGS provides better copy number resolution and the ability to identify rearrangements such as inversions that span a single exon of a tumor suppressor gene. With this type of inversion, there is no frameshift and the tumor suppressor gene is expressed at normal levels. Without isoform or splicing analysis, this change would be missed by IHC because it is still expressed at the protein level, even though the protein is inactive due to the missing amino acids [5]. We acknowledge that exome sequencing provides deeper coverage to identify coding mutations. Newer strategies may capture the best of both WGS and exome sequencing by include long insert shallow whole genomes and deep exome sequencing to capture complex rearrangements and achieve a higher sensitivity for capturing coding mutations.

Another challenge realized after the study was ongoing was the chain of custody handling for fresh biopsy samples. Because the initial tumors analyzed did not undergo DNA/RNA processing at a CLIA laboratory, the subsequent planned validation of targets from the same fresh sample could not be verified by a CLIA-certified test. Additionally, the approximate 2 weeks delay for CLIA validation coupled with a median time of 91 days for NGS results to become available was problematic. For patient 9's tumor, the KRAS (G12R) mutation was validated using a CLIA-certified test. For all but one patient (patient 2), a potentially druggable target with either a commercially available or investigational therapy was identified. For patient 11, WGS did not yield a target, and only WTS helped to identify druggable targets. The integration of epigenomics analysis could also factor as another layer towards facilitating identification of druggable targets with existing therapies.

Even if there are no technical or logical delays in NGS, the interpretation and application of this information to clinical practice is fraught with uncertainty. Not all SNVs identified by WGS will have a deleterious effect on protein function. So the mere identification of SNVs, even when validated in a CLIA-certified test, does not guarantee that targeting it will lead to stabilization or decrease in tumor burden. We must also recognize that intratumor heterogeneity and clonal evolution may complicate our devised treatment strategies for patients based on results from a single tumor biopsy [13], [14].

During the conduct of this study, we realized that with a median time-frame of 91 days to report of NGS results, patients with an excellent performance status and low tumor burden are more likely to have targets identified that can be acted upon. With improved efficiencies that decrease the time to get NGS results and reasonable costs, we can envision NGS can be applied more globally to advanced cancer patients. Even during the relatively short time that this study was enrolling, we observed significant improvements in sequencing analyses and lowered reagent costs. Others recently reported fairly impressive results with 27% response rate to available targeted therapies using more finite sequencing technology to identify druggable targets [15]. Overall, we demonstrate the feasibility of performing NGS technologies in a prospective manner in advanced cancer patients. We forecast that in the not to distant future NGS technologies will become more readily available for incorporation into routine care of advanced oncology patients.

## Conclusion

While the initial effort was a slower than anticipated process due to a variety of issues, we demonstrate that NGS can be utilized in clinical situations—whether this approach will lead to real and consistent patient benefit is yet to be proven.

## Supporting Information

File S1 **Supplementary Information.**
(DOCX)

Figure S1 **Patient 9 WTS data canonical maps.** This figure illustrates the top 20 canonical maps enriched in the WTS data for patient 9.
(TIFF)

Figure S2 **Role of stellate cells in progression of pancreatic cancer.** This figure illustrates the role of stellate cells in pancreatic cancer progression.
(TIFF)

Figure S3 **Development Regulation of epithelial to mesenchymal transition.** This figure illustrates the possible involvement of TGF-beta mediated epithelial to mesenchymal transition.
(TIFF)

Figure S4 **Selected canonical map for Patient 10: Cell cycle spindle assembly and chromosome separation.** This figure illustrates cell cycle spindle assembly and chromosome separation, including the upregulated and druggable target, aurora-B (AURKB).
(TIFF)

Figure S5 **Selected canonical map for Patient 10: EGF- and HGF-dependent stimulation of metastasis in gastric cancer.** This figure illustrates EGF- and HGF-dependent stimulation in gastric cancer metastasis. Alpha-6/beta-4 integrin components are upregulated and function in concert with MET to activate downstream signal transduction.
(TIFF)

Figure S6 **Patient 11 WTS data canonical maps.** This figure illustrates the top 20 canonical maps enriched in the WTS data for patient 11.
(TIFF)

Figure S7 **Tumor Copy Number Variations.** Copy number variation for Patients 2, 3, 4, 6, 7, 8, 9, 10, and 11. Y-axis is log2 fold-change (FC) and x-axis is chromosome and genomic position. Copy number gains are indicated with red (log2FC>0.75) and losses are indicated with green (log2FC<−0.75).
(TIFF)

Figure S8 **Key to Figures S2, S3, S4, S5.**
(TIFF)

Table S1

Table S2

Table S3
