# Supplementary material for: A Pilot Study Using Next-Generation Sequencing in Advanced Cancers: Feasibility and Challenges
Source: PLoS One. 2013 Oct 30;8(10):e76438. doi: 10.1371/journal.pone.0076438 (PMC3813699; doi:10.1371/journal.pone.0076438)
Supplement: File S1 — Supplementary Information. (DOCX) [file pone.0076438.s001.docx]

**Supplementary Information**

***Sample assessment***

Tumor samples were obtained and preserved as fresh frozen. Reference DNA was obtained from peripheral blood mononuclear cells. Direct visualization of samples collected from all patients (except patient 9 and 11) was obtained by two-ink frozen quality control procedure to estimate tumor content and extent of tissue heterogeneity by a board-certified pathologist (GH).

***Genomic DNA isolation***

Tissue was disrupted and homogenized in Buffer RLT plus (Qiagen AllPrep DNA/RNA Mini Kit), using the Bullet Blender^TM^, Next Advance, and transferred to a microcentrifuge tube containing Buffer RLT plus, and 1.6 mm stainless steel beads (patient 1) or 0.9 mm-2.0 mm RNase free stainless steel beads (patients 2 and 3). Blood leukocytes (buffy coat) were isolated from whole blood by centrifugation at room temperature and resuspended in Buffer RLT plus. All samples were homogenized, centrifuged at full speed, and lysates were transferred to the Qiagen AllPrep DNA spin column. Genomic DNA was purified following the manufacturer’s protocol. DNA was quantified using the Nanodrop spectrophotometer and quality was accessed from 260/280 and 260/230 absorbance ratios.

***RNA Isolation***

Tissue was disrupted and homogenized in Buffer RLT plus using the Bullet Blender, and transferred to a microcentrifuge tube containing Buffer RLT plus and 0.9 mm-2.0 mm RNAse free stainless steel beads. The tissue was homogenized in the Bullet Blender, and centrifuged at full speed. The supernatant was transferred to the Qiagen AllPrep DNA spin column. 70% ethanol was added to the flow-through and the mixture was applied to an RNeasy spin column. Total RNA purification was conducted as directed by the AllPrep DNA/RNA Mini Handbook. RNA was quantified using the Nanodrop spectrophotometer and quality was assessed using the Agilent Bioanalyzer.

Normal tissue or RNA samples were purchased as WTS controls. For those controls for which normal tissue was ordered, RNA isolation was performed. Below is a listing of the RNA control and source used for each patient:

Patient 2. Normal lung, RNA, Vendor: Agilent MVP

Patient 3. Normal pancreas, RNA, Vendor: Ambion FirstChoice

Patient 7. Normal lymph node, RNA, Vendor: Amsbio LLC

Patient 7. Normal skin, RNA, Vendor: Amsbio LLC

Patient 8. Normal ureter, Tissue, Vendor: Amsbio LLC

Patient 10. Normal kidney, Tissue, Vendor: Asterand

Patient 11. Normal lung, RNA, Agilen MVP

The same RNA control was used for patients 2 and 11. RNA was isolated from normal ureter and kidney using the Qiagen AllPrep Kit. RLT Buffer (Qiagen AllPrep Kit) and 0.9 – 2.0 mm stainless steel beads (Next Advance) were added to the tissue and the tissue was homogenized using the Bullet Blender (NextAdvance). RNA isolation was conducted using the AllPrep Kit manufacturer’s protocol.

***Whole genome library preparation***

3µg of genomic DNA from each sample was fragmented to a target size of 300-350 base pairs (bp). Overhangs in the fragmented samples were repaired to form blunt ends and adenine bases were ligated on. Diluted paired end Illumina adapters were then ligated onto the A-tailed products. Following ligation, samples were run on a 2% TAE gel to separate products. Ligation products at 300bp and 350bp were selected for each sample, isolated from gel punches, and purified. 2X Phusion High-Fidelity PCR Master Mix (Finnzymes; catalog#F-531L) was used to perform PCR to enrich for these products. Enriched PCR products were run on a 2% TAE gel and extracted. Products were quantified using Agilent’s High Sensitivity DNA chip (catalog#5067-4626) on the Agilent 2100 Bioanalyzer (catalog#G2939AA).

***Whole transcriptome library preparation***

All RNA samples were analyzed on the Agilent Bioanalyzer RNA 6000 Nano Chip to validate RNA integrity (RIN ≥ 7.0). 10ng of total RNA was used to generate whole transcriptome libraries for RNA sequencing. Using the Nugen Ovation RNA-Seq System (cat#7100-08), total RNA was used to generate double stranded cDNA, which was amplified using Nugen’s SPIA linear amplification process. Amplified cDNA was input into Illumina’s TruSeq DNA Sample Preparation Kit – Set A (cat#FC-121-1001) for library preparation. In summary, 1µg of amplified cDNA was fragmented to a target insert size of 300bp and end repaired. Samples were then adenylated and indexed paired end adapters were ligated. Ligation products were run on a 2% TAE gel and size selected at 400bp. Ligation products were isolated from gel punches and purified. Cleaned ligation products were input into PCR to enrich for libraries. PCR products were cleaned and quantified using the Agilent Bioanalyzer.

***Exome library preparation***

Exome libraries were prepared using 3µg of genomic DNA for each sample (tumor and germline for each patient). Genomic DNA was fragmented to an approximate target size of 150 to 200bp on the Covaris E210 using the following parameters: Duty cycle = 10%, Intensity = 5, Cycles/burst = 200, Time = 305 seconds, Bath temperature = 4°C. 100ng of each fragmented product was run on 2% TAE gel to verify fragmentation. Library preparation was performed using New England Biolab’s (NEB) NEBNext DNA Sample Prep Master Mix Kit, Illumina Multiplexing Oligonucleotide Kit, Agilent SureSelect Human All Exon 50Mb Kit, and Agilent Herculase II Fusion DNA Polymerase. End repair was performed using NEBNext End Repair Buffer (10X), End Repair Enzyme Mix, and the fragmented DNA samples. The reaction was incubated at 20°C for 30 minutes. End repair products were purified using AMPure XP beads: 180µL of resuspended beads were used for cleaning each sample, two 70% ethanol washes were performed, and samples were dried for 20 minutes at room temperature prior to resuspension in 44µL of warm EB. 42µL of cleaned end repaired samples are input into adenylation which is performed using NEBNext dA-tailing Buffer (10X) and NEBNext Klenow fragment (3’🡪5’ exo-). Reactions are incubated for 30 minutes at 37°C. Adenylated products are cleaned using AMPure XP beads as previously described but 90µL of beads are used for cleaning and the final samples are eluded with 15µL of water nuclease free water. 14µL of each adenylated sample is used for indexed adapter ligation. This step is performed using the NEBNext Ligation Buffer (5X), NEBNext T4 ligase, and Index PE adapter oligonucleotide mix from Illumina’s Multiplexing Oligonucleotide Kit. Reactions are incubated for 15 minutes at 20°C and cleaned using AMPure XP beads (90µL beads were used for cleaning each sample and final samples were eluded in 105µL EB). Enrichment PCR is next performed using InPE1.0 forward PCR primer (Illumina Multiplexing Oligonucleotide Kit), SureSelect Indexing Pre-cap PCR primer, Herculase II 5X reaction buffer, Herculase dNTP mix, and Herculase II polymerase. The following PCR program was used:

1. 98°C for 2 minutes

2. 98°C for 20 seconds

3. 65°C for 30 seconds

4. 72°C for 30 seconds

5. Cycle to step 2 five more times

6. 72°C for 5 minutes

7. 4°C hold

PCR products were purified using AMPure XP beads (90µL of beads were used for cleaning each sample and final samples were eluded in 40µL warm nuclease free water). Each sample is run on the Agilent Bioanalyzer using the Agilent DNA 1000 assay and quantified using the Qubit. 500ng of each sample is used for capture; an aliquot of each sample is adjusted to 147ng/µL in nuclease free water. From hybridization onwards, Agilent’s SureSelect Target Enrichment System for Illumina Paired-End Multiplexed Sequencing protocol (version 1.2) was followed. Final libraries are run on the Agilent Bioanalyzer.

***Paired end next generation sequencing***

Whole genome tumor and normal libraries, exome tumor and normal libraries for patients 6 and 8, and tumor RNA libraries were denatured using NaOH and diluted using Illumina’s HT1 buffer to prepare for clustering and paired end sequencing. Clusters were generated using Illumina’s cBot and HiSeq Paired End Cluster Generation Kits (catalog#PE-401-1001, PE-401-3001) and sequenced on Illumina’s HiSeq 2000 using Illumina’s HiSeq Sequencing Kit (catalog#FC-401-1001, FC-401-3001). The raw data has been deposited in the NIH Short Read Archive (SRA) (dbGap accession number is phs000657.v1.p1). The SRA Biosample accession numbers are as follows:

1. SAMN02228123 Pt2_WG_tumor
2. SAMN02228124 Pt2_WG_normal
3. SAMN02228125 Pt2_RNA_tumor
4. SAMN02228126 Pt3_WG_tumor
5. SAMN02228127 Pt3_WG_normal
6. SAMN02228128 Pt3_RNA_tumor
7. SAMN02228129 Pt4_WG_tumor
8. SAMN02228130 Pt4_WG_normal
9. SAMN02228131 Pt4_RNA_tumor
10. SAMN02228132 Pt6_WG_tumor
11. SAMN02228133 Pt6_WG_normal
12. SAMN02228134 Pt6_exome_tumor
13. SAMN02228135 Pt6_exome_normal
14. SAMN02228136 Pt6_RNA_tumor
15. SAMN02228137 Pt7_WG_tumor
16. SAMN02228138 Pt7_WG_normal
17. SAMN02228139 Pt7_exome_tumor
18. SAMN02228140 Pt7_exome_normal
19. SAMN02228141 Pt7_RNA_tumor
20. SAMN02228142 Pt8_WG_tumor
21. SAMN02228143 Pt8_WG_normal
22. SAMN02228144 Pt8_exome_tumor
23. SAMN02228145 Pt8_exome_normal
24. SAMN02228146 Pt8_RNA_tumor
25. SAMN02228147 Pt9_WG_tumor
26. SAMN02228148 Pt9_WG_normal
27. SAMN02228149 Pt9_RNA_tumor
28. SAMN02228150 Pt10_WG_tumor
29. SAMN02228151 Pt10_WG_normal
30. SAMN02228152 Pt10_exome_tumor
31. SAMN02228153 Pt10_exome_normal
32. SAMN02228154 Pt10_RNA_tumor
33. SAMN02228155 Pt11_WG_tumor
34. SAMN02228156 Pt11_WG_normal
35. SAMN02228157 Pt11_exome_tumor
36. SAMN02228158 Pt11_exome_normal
37. SAMN02228159 Pt11_RNA_control
38. SAMN02228160 normal_lung_RNA
39. SAMN02228161 normal_pancreas_RNA
40. SAMN02228162 normal_lymph_node_RNA
41. SAMN02228163 normal_skin_RNA
42. SAMN02228164 normal_ureter_RNA
43. SAMN02228165 normal_kidney_RNA

***Data analysis***

Raw sequence data in the form of .bcl files were generated by the Illumina HiSeq 2000. These data were converted to .qseq files, which were used to generate .fastq files. Fastq files were validated to evaluate the distribution of quality scores and to ensure that quality scores do not drastically drop over each read. Validated fastq files were aligned to the human reference genome (build 36) using the Burrows-Wheeler Alignment (BWA) tool for patients 2, 3, 4, 6, 7, 8, and 9, and build 37 for patients 10 and 11. Following alignment, .sai files were used to create .sam (sequence alignment map) files [[14](#_ENREF_14)], which were input into SAMtools to create binary sequence (.bam) files. PCR duplicates were flagged for removal using Picard (<http://picard.sourceforge.net>), and base quality scores were recalibrated using GATK (Genome Analysis Toolkit) [[15](#_ENREF_15)]. Mutation analysis was performed to identify SNPs, indels, and CNVs. Circos plots were generated for each patient to summarize results from all variant analyses (Supplementary Figures S1-3).

SNP (single nucleotide polymorphism) calling was performed using SolSNP (<http://sourceforge.net/projects/solsnp/files/SolSNP-1.01/>) and Mutation Walker, a tool developed in house and that incorporates variant discovery tools from GATK. SNPs that were called using both tools were compiled and visually examined for false positives to create a final filtered list of true SNVs (single nucleotide variants). Indel (insertion/deletion) calling was performed using GATK and a somatic indel detection tool developed in house. Copy number analysis was completed by determining the log2 difference (or fold-change (FC)) of the normalized physical coverage (or clonal coverage) for both germline and tumor samples separately across a sliding 2kb window of the mean using approaches described elsewhere (1).

WTS data was aligned against human reference genome (build 36) with TopHat 1.2 for all patients except for patients 11 and 12, who were aligned against build 37. WTS reads were only aligned against the autosomes and sex chromosomes. Mitochondrial DNA and annotations were removed from the genome and annotation references prior to alignment. Cuffdiff was used to identify differentially expressed genes and isoforms. Differential analysis was performed on FPKM (Fragments Per Kilobase of exon per Million fragments mapped) expression values calculated for gene and isoform.

***Enrichment Analysis for Patients 9, 10, and 11***

Enrichment analysis was performed using Thomson Reuters Metacore software tool (version 6.11). A master gene based file was compiled that containing WTS, CNV, and SNV data. This gene file was used to run the functional ontology enrichment workflow using the pathway map ontology. The default background list of all human genes was used for enrichment statistic calculation. Enrichment was performed using the pathway map category with the following statistical thresholds for significance p < 0.5 and FDR < 0.1. Maps were then visualized in software with individual genomic data files, one each for WTS, CNV and SNV, to delineate where single gene alterations were located on enriched maps.

***References***

1. Craig DW, Joyce JA, Kiefer JA, Aldrich J, Sinari S, et al. (2012) Genome and Transcriptome Sequencing in Prospective Metastic Triple-Negative Breast Cancer Uncovers Therapeutic Vulnerabilities. Mol Cancer Ther. 12(1):104-116.
