# Supplementary figures and images for: A Pilot Study Using Next-Generation Sequencing in Advanced Cancers: Feasibility and Challenges
Source: PLoS One. 2013 Oct 30;8(10):e76438. doi: 10.1371/journal.pone.0076438 (PMC3813699; doi:10.1371/journal.pone.0076438)

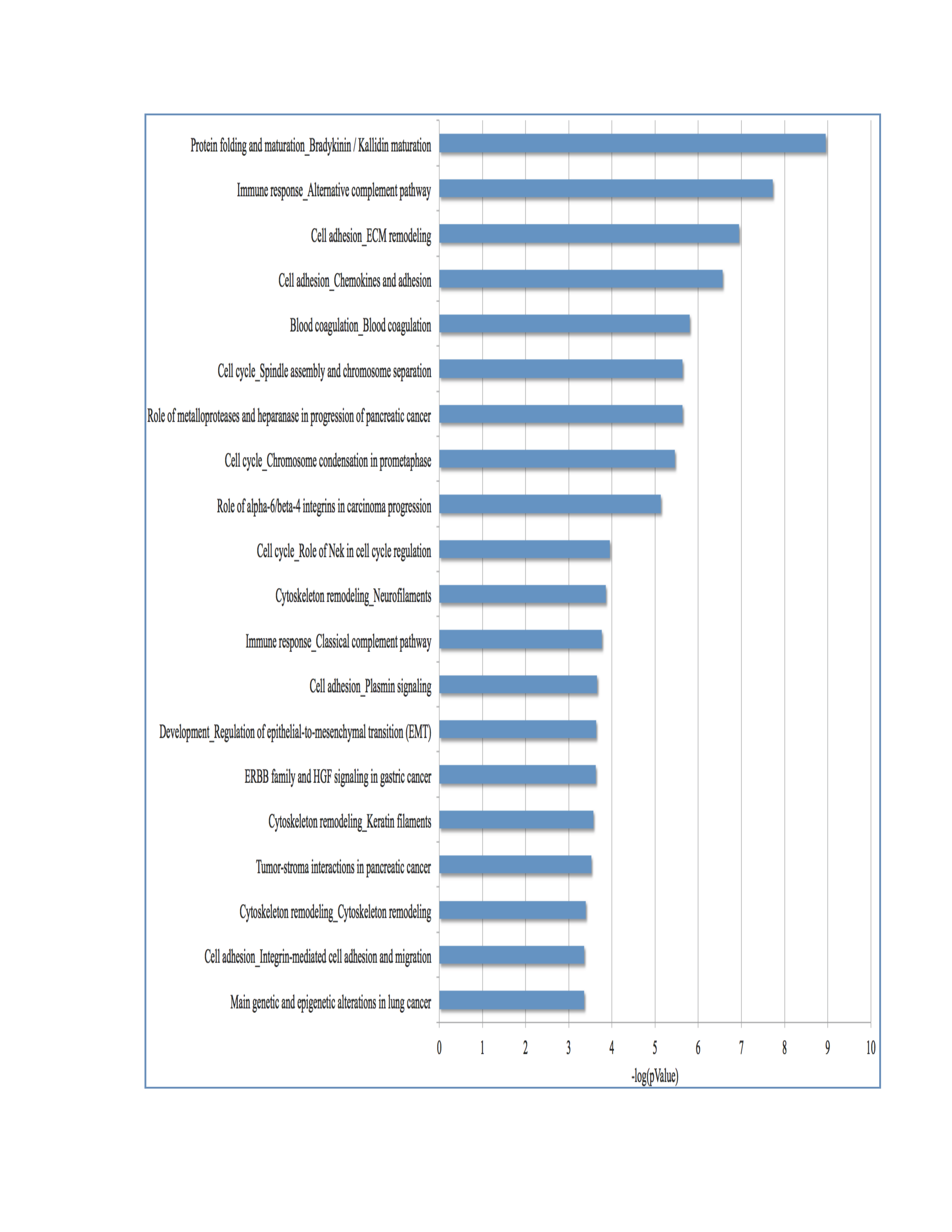

Supplement: Figure S1 — Patient 9 WTS data canonical maps. This figure illustrates the top 20 canonical maps enriched in the WTS data for patient 9. (TIFF) [file pone.0076438.s002.tiff]

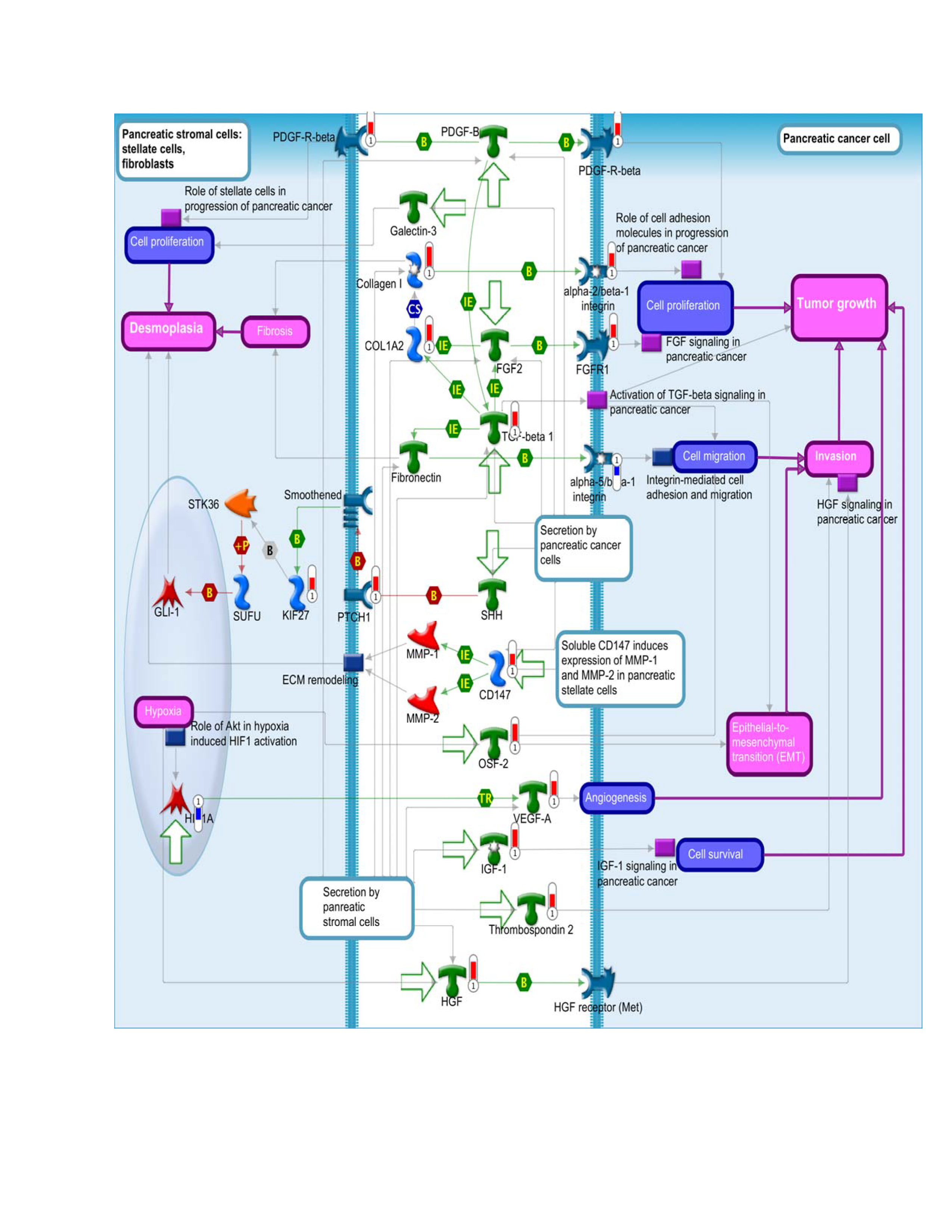

Supplement: Figure S2 — Role of stellate cells in progression of pancreatic cancer. This figure illustrates the role of stellate cells in pancreatic cancer progression. (TIFF) [file pone.0076438.s003.tiff]

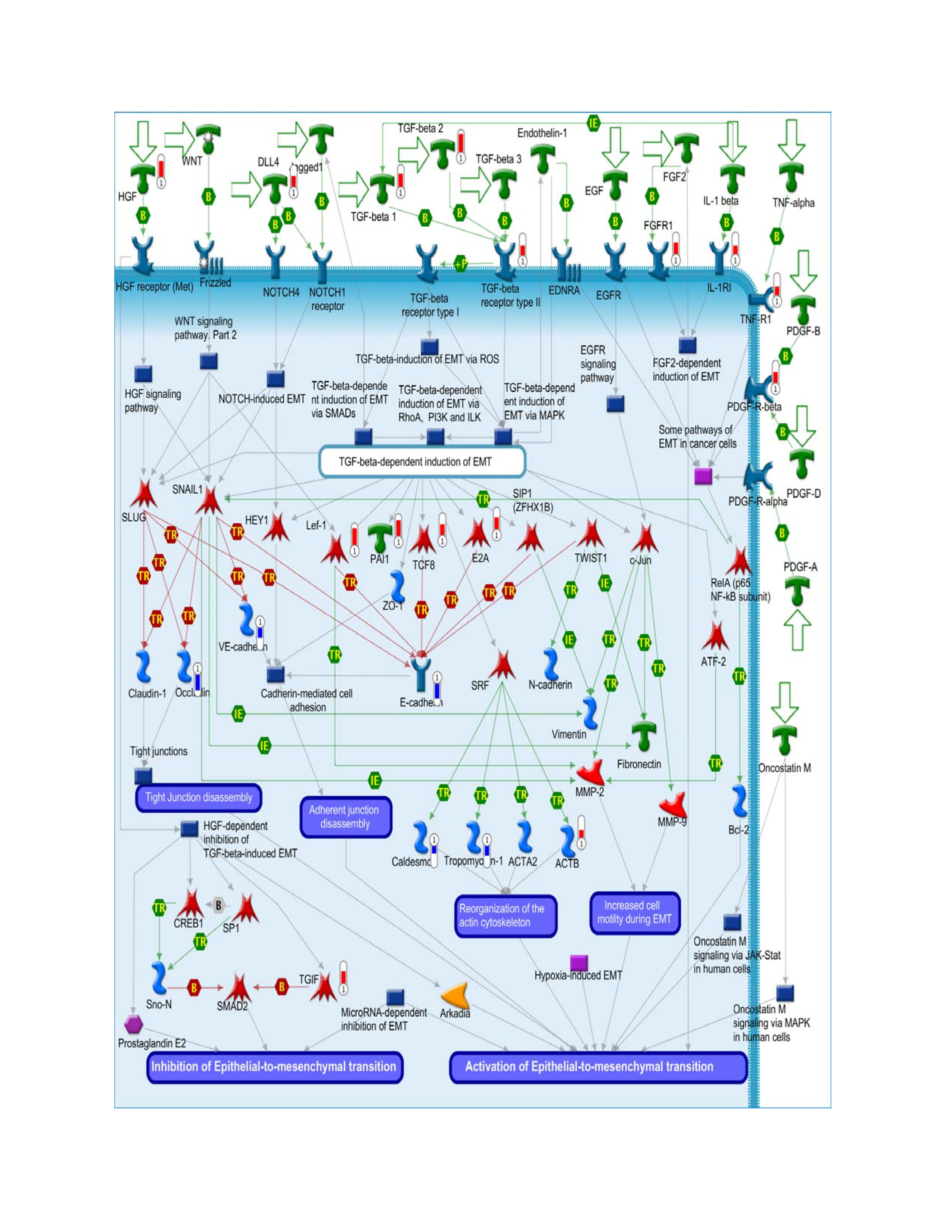

Supplement: Figure S3 — Development Regulation of epithelial to mesenchymal transition. This figure illustrates the possible involvement of TGF-beta mediated epithelial to mesenchymal transition. (TIFF) [file pone.0076438.s004.tiff]

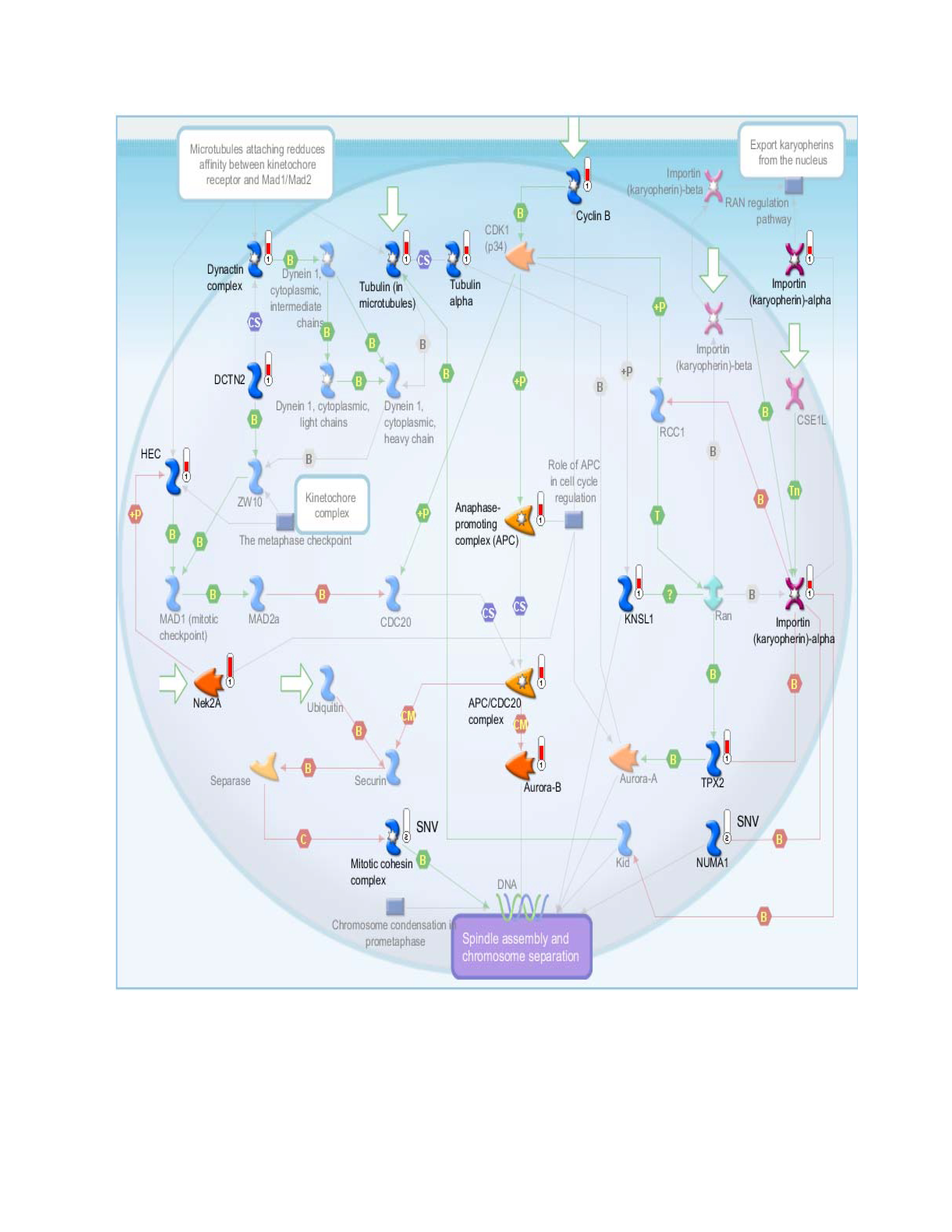

Supplement: Figure S4 — Selected canonical map for Patient 10: Cell cycle spindle assembly and chromosome separation. This figure illustrates cell cycle spindle assembly and chromosome separation, including the upregulated and druggable target, aurora-B (AURKB). (TIFF) [file pone.0076438.s005.tiff]

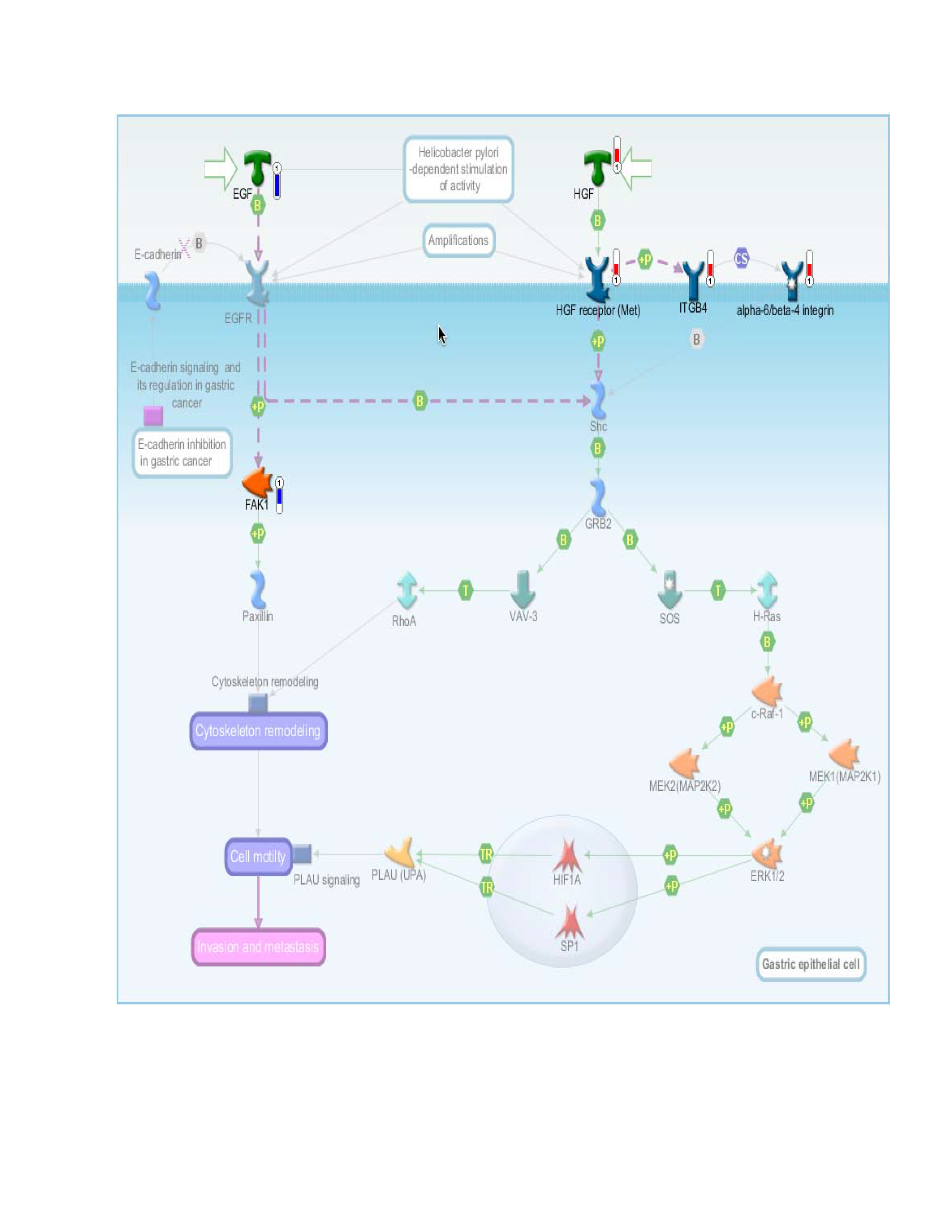

Supplement: Figure S5 — Selected canonical map for Patient 10: EGF- and HGF-dependent stimulation of metastasis in gastric cancer. This figure illustrates EGF- and HGF-dependent stimulation in gastric cancer metastasis. Alpha-6/beta-4 integrin components are upregulated and function in concert with MET to activate downstream signal transduction. (TIFF) [file pone.0076438.s006.tiff]

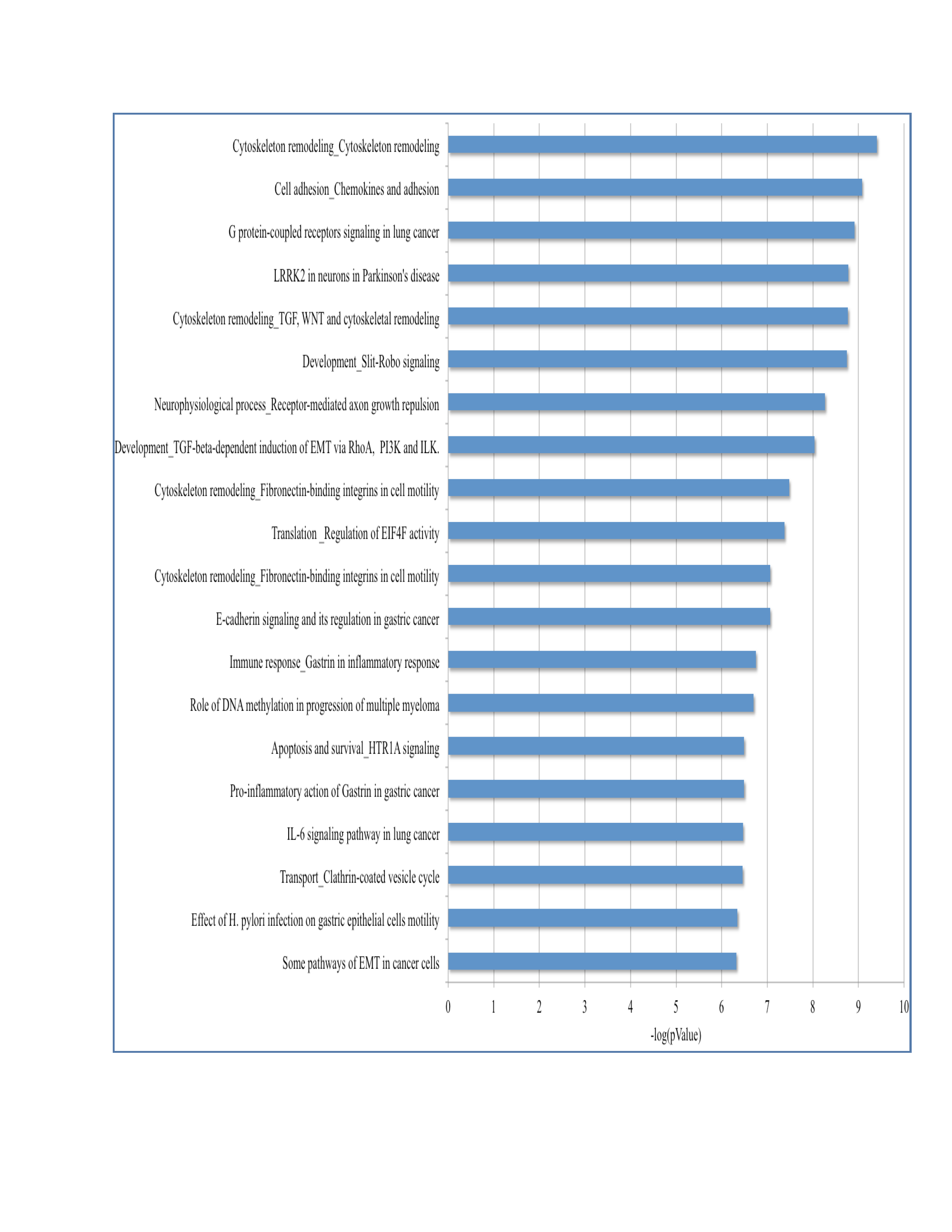

Supplement: Figure S6 — Patient 11 WTS data canonical maps. This figure illustrates the top 20 canonical maps enriched in the WTS data for patient 11. (TIFF) [file pone.0076438.s007.tiff]

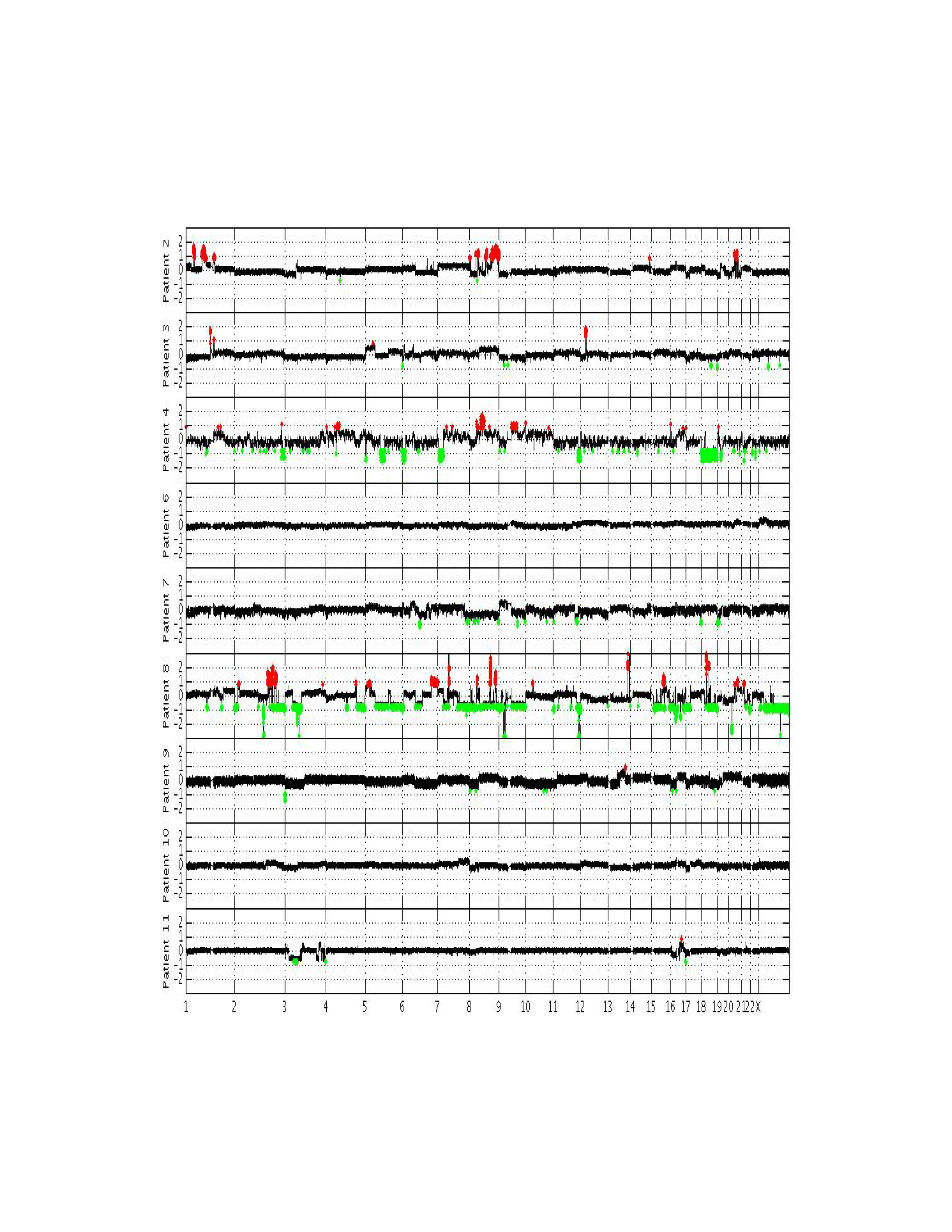

Supplement: Figure S7 — Tumor Copy Number Variations. Copy number variation for Patients 2, 3, 4, 6, 7, 8, 9, 10, and 11. Y-axis is log2 fold-change (FC) and x-axis is chromosome and genomic position. Copy number gains are indicated with red (log2FC>0.75) and losses are indicated with green (log2FC<−0.75). (TIFF) [file pone.0076438.s008.tiff]

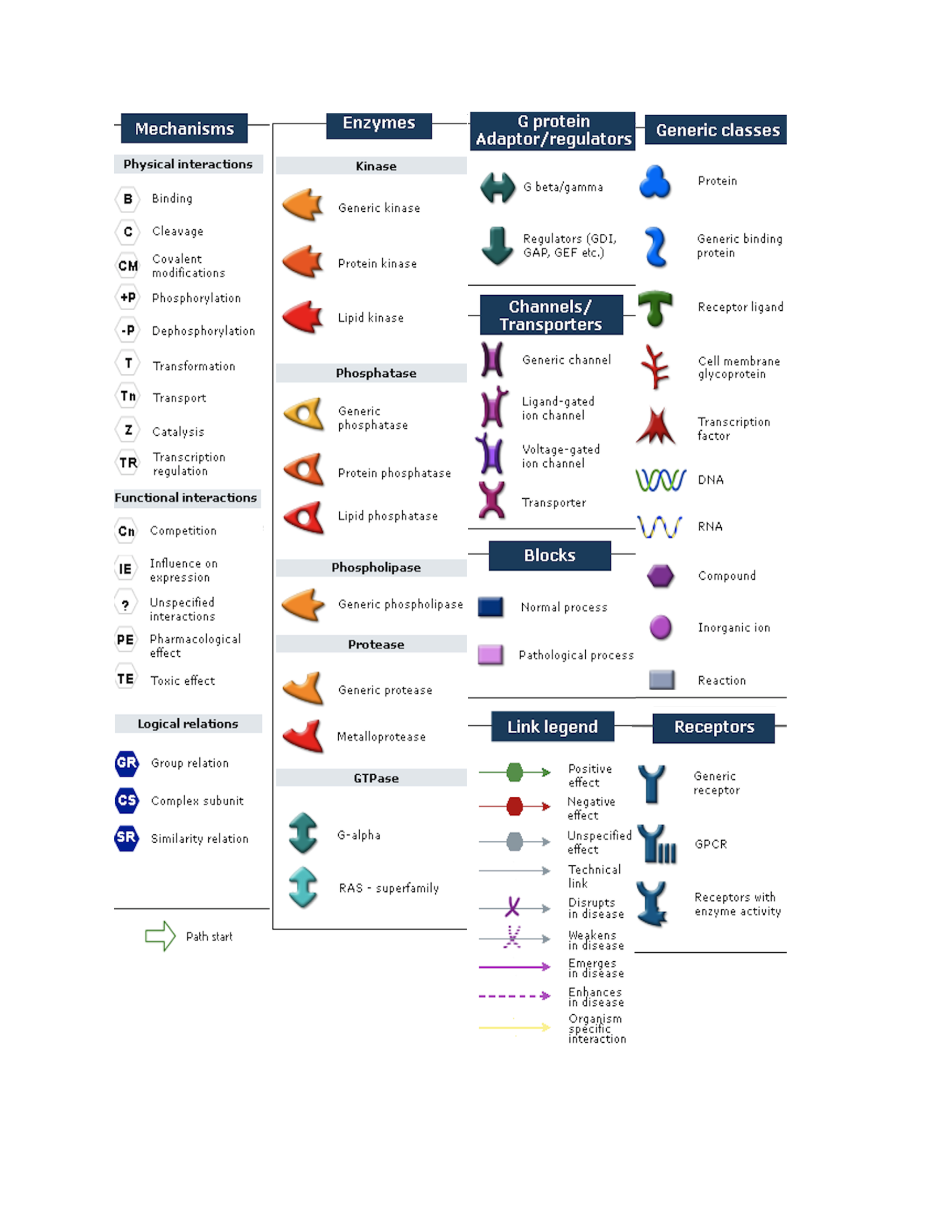

Supplement: Figure S8 — Key to Figures S2, S3, S4, S5. (TIFF) [file pone.0076438.s009.tiff]
